# Supplementary material for: Radiation-induced toxicity in rectal epithelial stem cell contributes to acute radiation injury in rectum
Source: Stem Cell Res Ther. 2021 Jan 15;12:63. doi: 10.1186/s13287-020-02111-w (PMC7811242; doi:10.1186/s13287-020-02111-w)
Supplement: Supplementary file 1 — Additional file 1. [file 13287_2020_2111_MOESM1_ESM.docx]

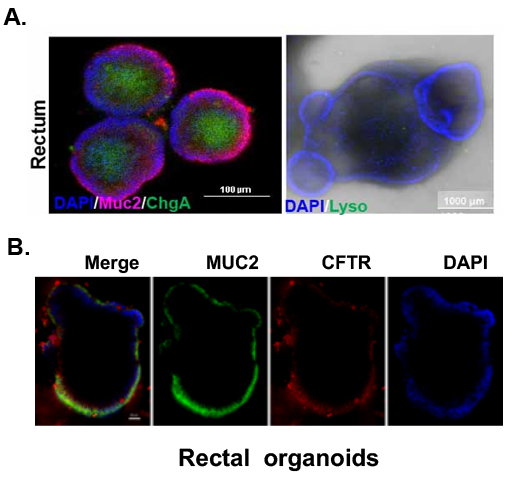


Supplement Figure 1

Fig1. Confocal microscopic image of the rectal organoid expressing markers predominant in rectal epithelium.

A) Representative image of mice rectal organoid co-expressing Muc2 (goblet cell marker) and ChgA (ChromograninA-enteroendocrine cell marker) (Left panel). Please note absence of paneth cell marker -Lysozyme expression predominantly expressed in small bowel and colon (right panel). B) Representative image of mice rectal organoid co-expressing Muc2 (goblet cell marker) and CFTR (cystic fibrosis transmembrane conductance regulator) protein specifically predominant in rectal epithelium.
